# The impacts of pretreatment on the fermentability of pretreated lignocellulosic biomass: a comparative evaluation between ammonia fiber expansion and dilute acid pretreatment

**DOI:** 10.1186/1754-6834-2-30

**Published:** 2009-12-04

**Authors:** Ming W Lau, Christa Gunawan, Bruce E Dale

**Affiliations:** 1 Department of Chemical Engineering and Materials Science, DOE Great Lakes Bioenergy Research Center, Michigan State University, Lansing, MI, USA

## Abstract

**Background:**

Pretreatment chemistry is of central importance due to its impacts on cellulosic biomass processing and biofuels conversion. Ammonia fiber expansion (AFEX) and dilute acid are two promising pretreatments using alkaline and acidic pH that have distinctive differences in pretreatment chemistries.

**Results:**

Comparative evaluation on these two pretreatments reveal that (i) AFEX-pretreated corn stover is significantly more fermentable with respect to cell growth and sugar consumption, (ii) both pretreatments can achieve more than 80% of total sugar yield in the enzymatic hydrolysis of washed pretreated solids, and (iii) while AFEX completely preserves plant carbohydrates, dilute acid pretreatment at 5% solids loading degrades 13% of xylose to byproducts.

**Conclusion:**

The selection of pretreatment will determine the biomass-processing configuration, requirements for hydrolysate conditioning (if any) and fermentation strategy. Through dilute acid pretreatment, the need for hemicellulase in biomass processing is negligible. AFEX-centered cellulosic technology can alleviate fermentation costs through reducing inoculum size and practically eliminating nutrient costs during bioconversion. However, AFEX requires supplemental xylanases as well as cellulase activity. As for long-term sustainability, AFEX has greater potential to diversify products from a cellulosic biorefinery due to lower levels of inhibitor generation and lignin loss.

## Background

Cellulosic ethanol, in comparison with first generation biofuels, is substantially more advantageous with regard to feedstock abundance and greenhouse gas reduction [1,2]. However, unlike the corn ethanol industry, lignocellulosic biomass processing requires higher severity pretreatments due to the inherent recalcitrance of plant material [3]. The selection of pretreatment method has a far-reaching impact on the overall process, including feedstock handling, biological conversions, and downstream processing [4]. The ability to generate steam and electricity from residual lignin is also crucial to maximize the economic profitability and environmental benefits of this industry [2].

Among potential pretreatment processes, dilute acid pretreatment and ammonia fiber expansion (AFEX) are regarded as promising candidates for large-scale cellulosic biofuel production. Dilute acid pretreatment has been extensively investigated and developed both in the laboratory and at pilot scale [5,6] to pretreat lignocellulosic biomass for fuel production. This pretreatment is a dry-to-slurry process which effectively hydrolyzes hemicellulose to soluble sugars in the liquor stream [7]. In contrast, AFEX is a dry-to-dry process at alkaline pH using anhydrous ammonia as the reaction catalyst. Although the macrostructure of the pretreated materials is preserved, AFEX reduces the degree of polymerization of cellulose and hemicellulose to increase enzyme accessibility for hydrolysis [8]. High sugar recoveries for corn stover (CS) can be achieved by both pretreatments, as shown by a previous comparative study [9].

However, a comprehensive comparison of pretreatments with regard to their impacts on important processing units is required. Lignocellulosic biomass is a complex material consisting primarily of cellulose, hemicellulose, lignin, and protein [10]. An ideal pretreatment should produce reactive biomass while minimizing the generation of inhibitory compounds that complicate bioconversions and downstream processes [4,11]. Furthermore, lignin and biomass nutrients must be preserved for coproduct generation.

In this report, we examine the impacts of these two pretreatments from an overall process perspective. Specifically, we evaluate the interactions of dilute acid pretreatment and AFEX with enzyme requirements, hydrolysate fermentability and lignin preservation. The microbial platform used for the pretreatment comparison involves *Saccharomyces cerevisiae *424A(LNH-ST) and *Escherichia coli *KO11. Comprehensive mass balances were also constructed around each pretreatment.

## Methods

### Corn stover

CS was supplied by the National Renewable Energy Laboratory (NREL, Golden, CO, USA). It was milled and passed through a 4 mm screen. The moisture content was approximately 7% (total weight basis). The milled CS was kept at 4°C for long-term storage. This CS contains 34.1% cellulose, 20.4% xylan, 3.3% arabinan and 2.3% protein on a dry weight basis.

### Dilute acid pretreated CS from pilot scale continuous (Sund) reactor at NREL

This dilute acid pretreatment was carried out as described previously [6]. Pretreatment was conducted at 190°C for a residence time of 45 to 75 s. The solids and sulfuric acid loading of the pretreatment were reported as 30% (w/w) and 0.048 g/g dry CS, respectively. The whole slurry from the reactor was used in this study.

### Pretreatment

#### AFEX

The AFEX pretreatment was performed in a 2.0 L pressure vessel (Parr Instruments, Moline, IL, USA). The reactor was equipped with thermocouples and a pressure sensor. AFEX on CS was conducted at 62.5% solids loading. The reactor was preheated to 100 to 110°C and prewetted CS (150 g dry CS + 90 g distilled water) was loaded into the vessel. The lid was bolted shut. Anhydrous ammonia (150 g) was preheated in a 500 mL stainless steel cylinder (Parker Instrumentation, Jacksonville, AL, USA) until the pressure reached 4.48 MPa (650 psi). Heated ammonia was then transferred into the reactor to initiate the reaction. The initial and final temperatures of the pretreatment were 130 ± 5°C and 110 ± 5°C, respectively. The reactor pressure was quickly released after 15 min through an exhaust value. AFEX-pretreated CS was then air dried in a fume hood overnight.

#### Bench scale dilute acid pretreatment

The dilute acid pretreatment was performed with a 1.0 L Parr reactor made of Hastelloy C (Parr Instruments, Moline, IL, USA) equipped with a thermocouple (Extech Instruments, Waltham, MA, USA) and a helical impeller (8.89 cm (3.5 inches)) on a two-piece shaft. The impeller was driven by a variable speed DC motor assembly (Parr Instruments). CS was presoaked in 1.0% w/v dilute sulfuric acid solution at 5.0% and 7.5% solids (w/w) overnight. The total weight of the pretreatment mixture was 800 g. The presoaked slurry was transferred into the reactor, which was then sealed and fitted to the impeller driver motor. The impeller speed was set at 150 rpm. The reactor was heated rapidly (within 2 min) to an internal temperature of 140 ± 2°C and maintained at 140 ± 2°C in a fluidized heating bath for 40 min. At the end of the reaction time, the reactor was cooled to below 50°C in a water bath. The combined severity factor of the pretreatment is 35.5. The diluted acid pretreated CS slurry was filtered through Whatman no. 1 filter paper. Details on the apparatus, experimental procedure and combined severity calculation are as described previously [7].

### Fermentation on water extract of soluble compounds from pretreated CS

#### Water extract/pretreatment liquor preparation

Four water extract/pretreatment liquors of pretreated CS were prepared for fermentation studies; they were prepared by (i) washing AFEX-CS pretreated CS, (ii) concentrating pretreatment liquor from dilute acid-CS pretreated CS, which was conducted at 5.0% solids loading in the bench scale reactor, (iii) concentrating/using pretreatment liquor from dilute acid-CS pretreated CS, which was conducted at 7.5% solids loading in the bench scale reactor and (iv) diluting pretreatment liquor from dilute acid-CS pretreated CS, which was conducted at 30.0% solids loading in a continuous pilot reactor (Sund). Solids-free water extracts were used for fermentation. The procedure to prepare the water extracts from different pretreatment was as follows.

**Figure 1:**
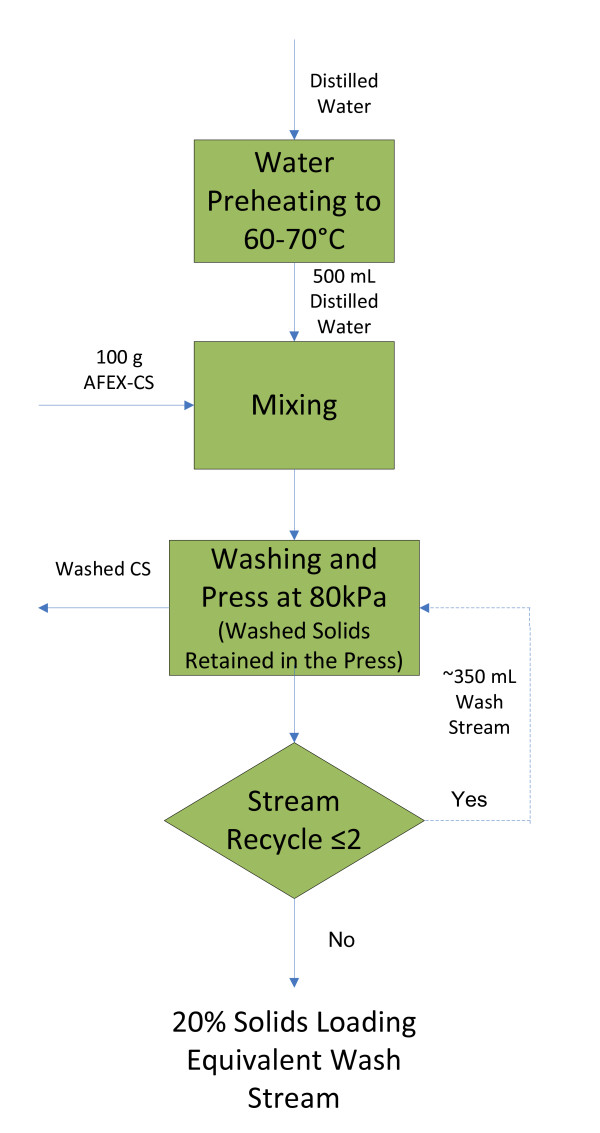
**Schematic describing the preparation of ammonia fiber expansion-corn stover water extract**.

AFEX-pretreated CS was washed with distilled water at a ratio of 1 g dry CS to 5 mL of water to produce a water extract (20% solids-loading equivalent). In each batch of washing, distilled water was preheated to 60 to 70°C and added to 100 g (dry weight equivalent) of AFEX-CS. The water content of the wetted AFEX-CS was reduced by using an in-house manufactured press. The washing was conducted in three cycles (that is, water extract from a previous cycle of washing was used for the next cycle of washing (Figure 1)). In the final cycle of washing, the moisture content of the washed AFEX-CS was reduced to 77 ± 3%. The AFEX-CS water extract was used for the fermentation.

Pretreatment liquor (hemicellulose hydrolysate) from the dilute acid pretreatment stage was used as the water extract. Hemicellulose hydrolysates prepared from 5% and 7.5% solids loading during bench scale dilute acid pretreatment were neutralized using KOH to pH 7.0 and concentrated to 20% solids-loading equivalent (1 g input CS in 5 mL liquid) through rotary evaporation under vacuum at 75°C. Xylose concentration was used as the indicator for the concentration factor achieved during the evaporation.

Where [Xyl], f and i denote for concentration of xylose, final and initial condition, respectively.

For CS from the Sund reactor, distilled water was added so that the mixture contained 5 mL of liquid to 0.51 g of dry water-insoluble pretreated CS. The diluted slurry was mixed by rigorous shaking and centrifuged at 6 000 *g*. The supernatant was at 20% solids-loading equivalent. No mass balance around Sund pretreatment was made available, therefore it was assumed that the percentage of input CS remaining as water-insoluble solids after the pretreatment in the Sund reactor was the same as that of bench scale dilute acid pretreatment (that is, 51%).

#### Seed culture preparation

Seed cultures of *E. coli *KO11 and *S. cerevisiae *424A(LNH-ST) were prepared in 100 mL of complex media YEP_GX (5 g/L bacto yeast extract + 10 g/L bacto peptone + 30 g/L glucose + 20 g/L xylose) by inoculating frozen (-80°C) culture stock at an initial cell density of 0.1 unit optical density (OD) 600 nm using a UV/Vis spectrophotometer (Beckman Coulter, DU720, Brea, CA, USA). The culture temperatures and periods for KO11 and 424A(LNH-ST) were 37°C, 18 h and 30°C, 18 h, respectively. The cultures were conducted under microaerophilic conditions and mixed at 150 rpm agitation. The grown cells were used to initiate fermentations

### Fermentation procedure

#### Microplate fermentation

Fermentations of *E. coli *KO11 and *S. cerevisiae *424A(LNH-ST) in wash streams of 7.5% and 15.0% solids-loading equivalent of the three types of pretreated CS were conducted in 24-well cell culture microplates (BD Falcon #353047, San Jose, CA, USA). The media were supplemented with wash stream, yeast nitrogen base (YNB) with ammonium sulfate (MP Biomedicals, lot no.s 4027512-119914, Solon, OH, USA), glucose and xylose in appropriate buffer (50 mM) at final concentrations of 16.7 g/L, 9 g/L and 35 g/L, respectively. Distilled water was added to dilute the wash streams to 7.5% and 15.0% solid loading equivalent. Chloramphenicol (50 mg/L) was added to reduce the risk of contamination.

Each well contained 2.0 mL media and a glass bead was added (6 mm in diameter) to aid stirring. Seed cultures were prepared as described above and the microplate cell culture was initiated at OD 600 nm of 0.5. The microplate was sealedand fixed on the microplate clamp system (Applikon Inc, Springfield, IL, USA) in an incubator shaker (150 rpm). An opening (about 1 mm diameter) was made on the seal to vent CO_2 _produced. The initial pH for *E. coli *KO11 was at 7.0 and at 5.5 for *S. cerevisiae *424A(LNH-ST). The incubation temperature was the same as seed culture conditions. The fermentations were conducted for a designated period (*E. coli *KO11, *S. cerevisiae *424A(LNH-ST): 24 h). Cell density was measured using a spectrophotometer at an OD of 600 nm. Sugars and fermentation products were analyzed using a high-performance liquid chromatography (HPLC) system with a Biorad Aminex HPX-87 H column as described previously [12]. Error bars shown in the results are standard deviations of triplicates.

#### Shake flask fermentation

Fermentations of KO11 and 424A(LNH-ST) were further conducted in 250 mL shake flasks with a 70 mL working volume capped with a rubber stopper, pierced with a needle to vent CO_2 _formed during fermentation. Wash stream from AFEX and dilute acid pretreatment were supplemented with 1 g/L yeast extract and 2 g/L peptone with 3-(*N*-morpholino)propanesulfonic acid (MOPS)/phosphate buffer. Sugar levels were adjusted to about 10 g/L glucose and 50 g/L xylose. Final solids-loading equivalents were 7.5%. Inoculum was added to achieve an initial cell density of 0.1 OD 600 nm. Fermentations of *E. coli *KO11 and *S. cerevisiae *424A(LNH-ST) were conducted at 37°C and 30°C, respectively, at 150 rpm agitation. KO11 fermentation was pH adjusted every 24 h using 6 M KOH to pH 7.0. 424A(LNH-ST) fermentation was not pH adjusted because the pH was stable at between 5.2 to 5.5 throughout the fermentation. Fermentation samples were taken at designated points throughout the 120 h culture.

### Enzymatic hydrolysis

#### Enzymatic hydrolysis of water-insoluble solids of the pretreated CS

To prepare water-insoluble materials, pretreated CS from both pretreatments was washed with distilled water at a ratio 1 dry g (input CS to pretreatment) to 50 mL of water. For bench scale dilute acid pretreated CS, the designated amount of distilled water was poured into a filter system with Whatman filter paper (no. 4) under vacuum. The solids remaining on the filter paper were dried under vacuum at 60°C. For AFEX-pretreated CS, the washing was achieved in two stages: (1) incubation at 250 rpm, 50°C for 24 h at 5% solids-loading equivalent and (2) two cycles of centrifugation at 6 000 *g*. After each cycle of centrifugation, the supernatant was decanted through the filter system. The total weight of water-insoluble solids was measured and the carbohydrate content of the solids was analyzed using NREL protocol LAP-002.

The water-insoluble materials were enzymatically hydrolyzed using either (i) cellulase mixtures or (ii) cellulase + hemicellulase mixtures at pH 4.8, 50°C for 144 h. The cellulase mixture consisted of Spezyme CP (86.7 mL/kg CS; 15 FPU/g cellulose) and Novozyme 188 (87.5 mL/kg CS; 64 *p*NPGU/g cellulose). The hemicellulase mixture was Multifect Xylanase (12.7 mL/kg CS) and Multifect Pectinase (12.7 mL/kg CS). The spectra of activities for the commercial enzymes were as reported [13]. The Spezyme and Multifect enzymes were obtained from Genencor Inc. (Palo Alto, CA, USA) and Novozyme 188 was purchased from Sigma-Aldrich Co. (St Louis, MO, USA). Enzymatic hydrolysis was conducted at 5.1% glucan loading. Glucose and xylose in both monomeric and oligomeric forms were measured. Error bars shown are standard deviations of triplicates.

### Mass balance construction

#### Carbohydrate mass balance around pretreatment

After pretreatment, the pretreated solids from both AFEX and dilute acid pretreatment were washed with water at a ratio of 1 g input biomass to 50 mL of water. The total mass and dry matter content (%) of the input and output materials around the pretreatments were recorded. The volumes of the wash streams were recorded. The glucan and xylan content of the dry matters were analyzed using NREL protocol LAP-002. Both monomeric and oligomeric (LAP-014) sugars of the wash stream from AFEX-pretreated CS and hemicellulose hydrolysate from dilute acid pretreated CS were analyzed. The total anhydrous equivalent of glucose and xylose were calculated for input and output around both pretreatments. The percentage of carbohydrate conserved was calculated as follows.

#### Klason lignin mass balance around pretreatment

The dry matter mass of the input and output materials around pretreatment and enzymatic hydrolysis were recorded. The total percentage of Klason lignin in the dry matters before and after pretreatment was analyzed using NREL protocol LAP-002. The final acid concentration, temperature and residence time for the assay was 4% sulfuric acid, 121°C and 60 min, respectively. The Klason lignin content was calculated by multiplying the total dry matter by the percentage of Klason lignin.

#### Residual solids analysis and heat value estimation

After enzymatic hydrolysis at 5.1% glucan loading, unhydrolyzed solids were separated by centrifugation, washed twice using distilled water, and dried under vacuum at 55°C. The total dry weight was recorded. Glucan and xylan in the unhydrolyzed solids was analyzed using NREL protocol LAP-002. Residual non-carbohydrate solids and their heating value were estimated as described below.

This is done by assuming 90% of the total residual solids is lignin and the rest of 10% has negligible heat value. The heat value of lignin used (25.4 kJ/g) was as reported [14].

## Results

### Sugar and lignin preservation during AFEX and dilute acid pretreatment

**Figure 2:**
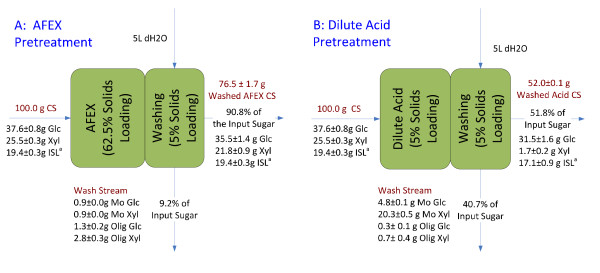
**Mass balance comparison between ammonia fiber expansion (AFEX) and dilute acid pretreatment on corn stover**. (a) AFEX pretreatment and (b) dilute acid pretreatment. AFEX and dilute acid pretreatment were conducted at 62.5% and 5.0% solids loading, respectively. Washing of pretreated corn stover from both pretreatment was carried out at 5.0% solids loading based on input materials to the pretreatment. ISL = insoluble lignin (Klason lignin). ^a^The assay condition for the insoluble lignin measure was 4% final H_2_SO_4 _concentration, 121°C, 60 min.

AFEX pretreatment on CS at 62.5% solids loading preserved all the carbohydrates. Nearly 10% of the AFEX-pretreated CS carbohydrate was water soluble, of which two-thirds was monomeric or oligomeric xylose (Figure 2a). However, 13% of the xylose sugar was degraded in the dilute acid pretreatment at 5% solids loading. About half of the total input solids were solubilized in the acid solution. While 59% of the total remaining solids after dilute acid pretreatment are glucan, the xylan content was reduced to about 3% (Figure 2b). In all, 42% of the total output sugars from dilute acid pretreatment were water soluble, predominantly in monomeric forms. The concentration of the total sugars in the acid liquid stream was 14 g/L.

With regard to the Klason lignin content (at assay condition: 4% sulfuric acid, 121°C, 60 min), AFEX pretreatment did not remove Klason lignin from the solids. For dilute acid pretreatment, 12% of the Klason lignin was removed.

### Cell growth and fermentation in soluble extract of the pretreated CS

**Figure 3:**
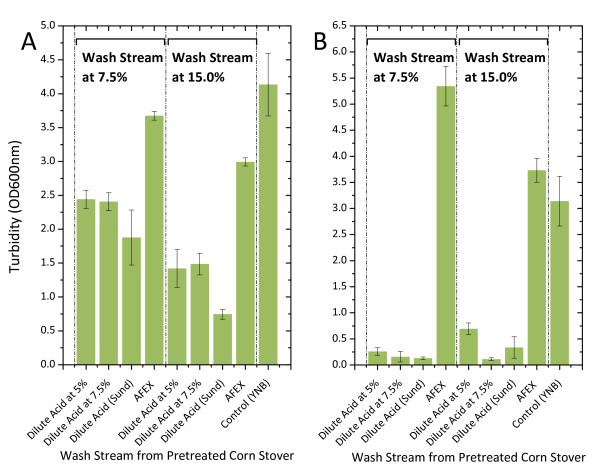
**Cell density of (a) *Saccharomyces cerevisiae *424A(LNH-ST) and (b) *Escherichia coli *KO11 after 24 h of fermentation in yeast nitrogen base (YNB)-supplemented wash streams from dilute acid pretreatment at different solids loading (5%, 7.5%, 30% (Sund)) and ammonia fiber expansion (AFEX) pretreatment**. Fermentation was conducted in 24-well microplates at 2.0 mL working volume with an initial cell density of 0.1 unit of optical density (OD) 600 nm. The solids-loading equivalent of the wash streams tested was 7.5% and 15.0%.

Categorically, the wash stream from AFEX-treated CS exhibited significantly higher fermentability with regards to cell growth and glucose and xylose consumption in both *S. cerevisiae *424A(LNH-ST) and *E. coli *KO11. Remarkably, the wash stream from dilute acid pretreatment inhibited the growth of KO11 completely in both 7.5% and 15% solids loading (Figure 3) over the tested fermentation period (24 h) (Figure 3). Comparing different dilute acid pretreatment approaches, the wash stream of pretreated CS from the Sund reactor was more inhibitory than the bench scale, low solid-loading pretreatments. The cell density of 424A(LNH-ST) in the Sund-CS wash stream was about half of that of acid pretreated CS at laboratory bench scale.

**Figure 4:**
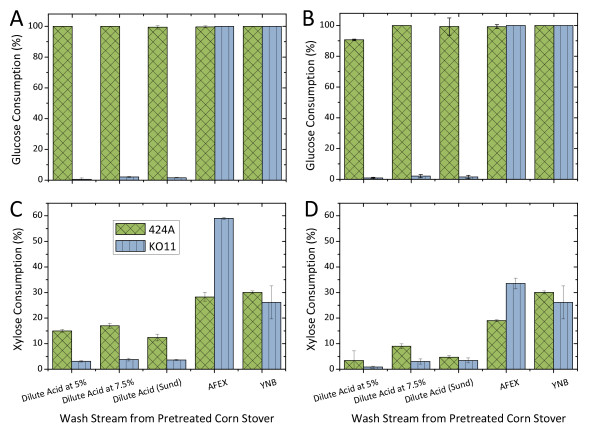
**Sugar consumption after 24 h of fermentation in yeast nitrogen base (YNB)-supplemented wash streams from dilute acid pretreatment at different solids loading (5%, 7.5%, 30% (Sund)) and ammonia fiber expansion (AFEX) pretreatment in wash stream at 7.5% solids loading equivalent (a, c) and 15.0% solids loading equivalent (b, d)**. Fermentation was conducted in 24-well microplates at 2.0 mL working volume with an initial cell density of 0.1 unit of optical density (OD) 600 nm. Initial glucose and xylose was 9 ± 1 g/L and 35 ± 2 g/L, respectively.

In contrast, all tested AFEX-CS wash streams were high fermentable. In essence no inhibitory effect on cell growth was observed. Fermentations of AFEX-treated material performed similarly to that of yeast nitrogen base (YNB, 13.7 g/L). In the case of KO11 fermentation, xylose consumption in AFEX-CS wash stream (7.5% solids-loading equivalent) was twofold higher than that of YNB. Complete glucose (8 to 10 g/L) fermentation was achieved regardless of pretreatments by *S. cerevisiae *(Figure 4a, b). While better xylose fermentation was achieved in KO11 than 424A(LNH-ST) in AFEX-CS wash stream, the opposite trend was observed in dilute acid-CS wash stream due to the inhibitory nature of the wash stream and the strain robustness toward the inhibitors.

**Figure 5:**
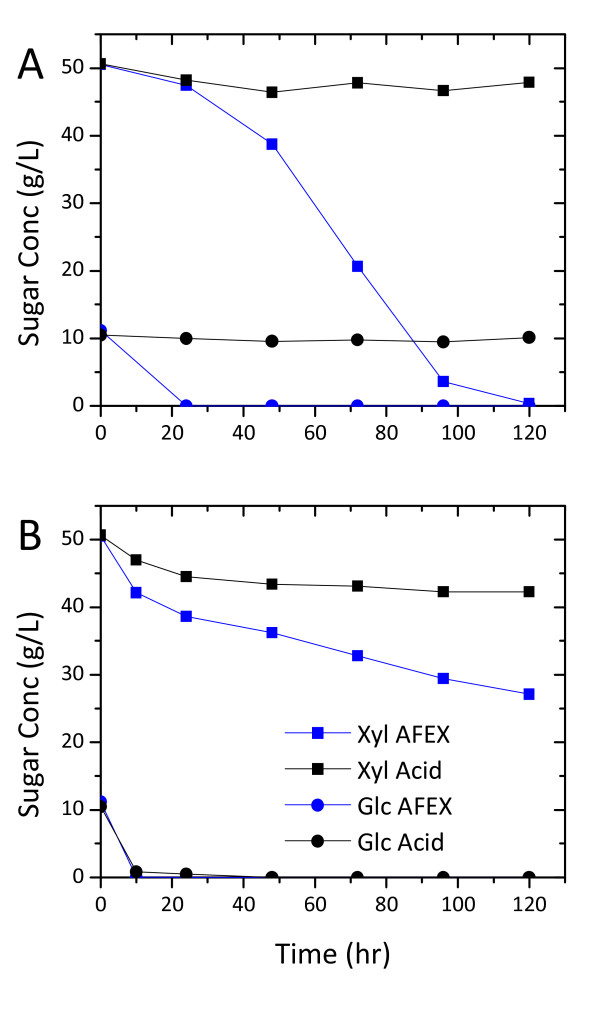
**Time course of fermentation of wash stream from 7.5% solids loading equivalent of ammonia fiber expansion (AFEX) and dilute acid pretreated corn stover**. Fermentation was initiated at 0.1 unit optical density (OD) 600 nm and the wash streams were supplemented with 1 g/L yeast extract and 2 g/L peptone.

The time courses of KO11 and 424A(LNH-ST) fermentations in both wash streams also showed similar trends as the microplate fermentations regarding strain robustness of 424A(LNH-ST) and better xylose fermentation of KO11 in AFEX-CS wash stream (Figure 5). At the low initial cell density tested, KO11 could not grow in the wash stream from the acid pretreated CS over the fermentation period.

### Enzymatic hydrolysis of washed pretreated solids

**Figure 6:**
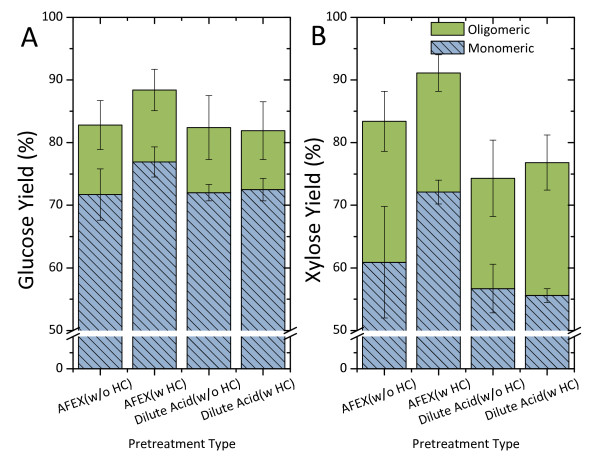
**Enzymatic hydrolysis yield on water-insoluble ammonia fiber expansion (AFEX)-pretreated corn stover and dilute acid pretreated corn stover at 5.1% glucan loading, pH 4.8 and 50°C**. Note: the xylan content in water-insoluble dilute acid pretreated corn stover is very low (3%).

Enzymatic hydrolysis at 5.1% glucan loading of all tested washed solids from both pretreatments achieved similar total glucose yields at 82% when the cellulase-only mixture was used. However, AFEX achieved 6% higher glucose yield when both cellulase and hemicellulase was added (the difference was within the margin of error) (Figure 6). The added hemicellulase mixture also improved xylose yield in AFEX improved from 83% to 91%. In contrast, the hemicellulase mixture does not affect sugar yields in dilute acid pretreated solids. This is probably due to the low xylan content (about 3%) in the solids. The proportions of glucose and xylose oligomers to the total sugars in the hydrolysates from both pretreatments was about 12.5% and 25.0%, respectively (Figure 6).

### Energy content of non-carbohydrate residual solids

**Figure 7:**
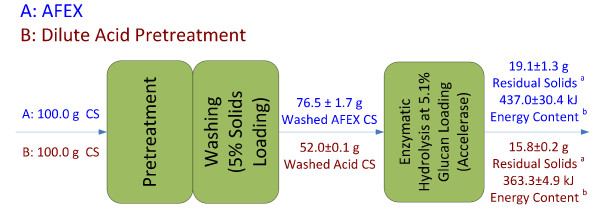
**Mass balance and energy content for non-carbohydrate insoluble solids after pretreatment and washing and enzymatic hydrolysis**. ^a^Residual solids = (recorded total dry solids left unhydrolyzed) - (dry glucan and xylan in the solids). ^b^Energy content = 0.9 × residual solids (g) × 25.4 kJ/g. This is done by assuming 90% of the total residual solids are lignin and the remaining 10% has negligible heat value. The heat value of lignin used (25.4 kJ/g) was as reported.

Non-carbohydrate residual solids from AFEX and dilute acid pretreatment and hydrolysis were 19.1 g and 15.8 g per 100 g of untreated CS, respectively. Based on the calculation method listed, biomass-processing technology based on AFEX pretreatment is able to generate 737 kJ/kg more energy from the residual solids than that of dilute acid pretreatment. About 23.2% (AFEX) and 19.3% (dilute acid) of the heating value in the untreated CS remained in the non-carbohydrate residual solids (Figure 7).

## Discussion

### Dilute acid pretreatment reduces maximum possible product yield by 10%

The viability of a commercial process is highly dependent on overall process yield. Hence, efforts to increase ethanol yield per unit mass of biomass (CS) at a given product titer deserve the highest priority. In this regard, AFEX preserves all carbohydrates while effectively increasing the susceptibility of the pretreated CS to hydrolytic enzymes. Unlike AFEX, acid-catalyzed pretreatment hydrolyzed hemicellulose almost completely. Monomeric pentoses are further degraded to byproducts such as furfural under acid treatment conditions. In our investigations, about 13% of xylan was lost through chemical degradation. However, a greater degree of degradation (20% to 30%) was reported at a higher solids loading of dilute acid pretreatment [6]. This reduces the maximum product yield by 10%. In any mature chemical process for commodities, raw material is the dominant factor in the processing costs [15]. Therefore, selection of a pretreatment that highly preserves plant carbohydrates is critical for long-term success in this industry.

### Pretreatment dictates the fermentability of pretreated biomass

Apart from the preservation of carbohydrate, an ideal pretreatment reduces the generation of inhibitory degradation compounds. AFEX-pretreated CS is highly fermentable using both bacteria and yeast. In certain cases, the soluble fraction of AFEX-pretreated CS has been shown to be beneficial to microbial growth [16]. In contrast, CS hydrolysate from dilute acid pretreatment is substantially more inhibitory. The nitrogenous (amides and amines) reaction products formed during ammonia-lignocellulose reactions are generally non-inhibitory toward microbial growth. These degradation products would otherwise be organic (aliphatic and phenolic) acids in acid-catalyzed reactions [17]. Fermentation at higher initial cell density, nutrient supplementation and/or detoxification are likely needed to alleviate or overcome their inhibitory effects of acid pretreatment [18,19].

### Pretreatment determines feasible biomass-processing configurations

Due to the nature of pretreatment, particularly with respect to the degree of hemicellulose solubilization, inhibitor generation and nutrient preservation, different biomass-processing strategies that maximize the advantages of each pretreatment should be exploited. Dilute acid pretreatment is reported to be well suited for softwood materials [20] and effectively hydrolyze hemicellulose, eliminating the need for hemicellulases during enzymatic hydrolysis. Nevertheless, the hemicellulase stream is inhibitory toward enzymes and microorganisms. Therefore, separation of solids and the hemicellulose stream as previously proposed [21] is essential to minimize the adverse effects of the inhibitors from the bioconversion of the remaining solids. However, important technical issues need to be solved in a cost-effective fashion, including (i) separation of solids and liquid with low fresh water use and (ii) effective fermentation of the hemicellulose stream at high sugar concentration without significant conditioning.

AFEX-centered biomass processing can be performed in a straightforward manner where the pretreated biomass (cellulose and hemicellulose) can be converted to ethanol after enzymatic hydrolysis and fermentation without washing or stream separation [16]. In this report, washing was done in AFEX-pretreated CS to establish a basis for comparison. Due to high fermentability of AFEX-pretreated biomass, washing, nutrient supplementation and high initial cell density are not required during the fermentation stage [16]. In comparison to dilute acid pretreatment, a relatively large portion of oligomeric xylose is present in AFEX hydrolysate. Exploitation of hemicellulase-secreting strains such as *Thermoanaerobacterium saccharolyticum *to biologically process AFEX-pretreated materials could address this issue without added cost of hemicellulase [22].

### AFEX enhances coproduct generation and diversity

Reduction in greenhouse gas emissions by cellulosic ethanol E85 relative to petroleum gasoline is projected to be 68% to 102%, and this is largely due to the heating value of residual solids (primarily lignin) to generate steam or electricity as a coproduct [2,23]. Our results indicate that AFEX-centered cellulosic technology is expected to have about 17% more available energy from the insoluble lignin residue compared to dilute acid. This also implies that the selection of pretreatment directly affects the magnitude of environmental benefits brought about by a cellulosic ethanol plant beyond the direct impact of the pretreatment process. Nevertheless, a definitive conclusion on the impact of different pretreatments on various environmental benefits can only be made after careful life cycle analysis based on these experimental data.

Lignin removal is a function of severity in terms of acid concentration, temperature and residence time [24], and part of the solubilized lignin can be recovered [25]. However, the recovery process will inevitably increase the processing cost relative to a production process where lignin is preserved in the solid residue.

## Conclusion

AFEX, a dry-to-dry pretreatment process, completely preserves Klason lignin and carbohydrate. In comparison, 13% of the xylan was degraded to byproduct and 12% of the Klason lignin was not preserved in the dilute acid pretreated CS. Categorically, streams resulting from AFEX-CS displayed significantly better fermentability than those from dilute acid. While dilute acid pretreatment eliminates the need for hemicellulolytic enzymes for hydrolysis, AFEX-centered cellulosic technology simplifies production steps, reduces the requirement for nutrient supplementation, increases the diversity of coproducts and potentially enhances the environmental benefits beyond the direct impact of the pretreatment processes. This is largely due to the nature of the pretreatment chemistries, which reduces inhibitory degradation compound generation and preserves lignin in solid residues while being effective in overcoming biomass recalcitrance that increases the susceptibility of biomass constituents (carbon or nitrogen sources) for digestion.

## Competing interests

The authors declare that they have no competing interests.

## Authors' contributions

MWL designed and carried out experiments, analyzed results, and wrote the manuscript. CG carried out experiments and analyzed results. BED analyzed results and reviewed the manuscript. All authors read and approved the final manuscript.
